# Validity of the BOT-2 Short Form for Korean School-Age Children: A Preliminary Study

**DOI:** 10.3390/children11060724

**Published:** 2024-06-13

**Authors:** Deukgeun Yoon, Dabin Choi, Misun Kim, Seokyeon Ji, Yoo-Sook Joung, Eun Young Kim

**Affiliations:** 1 Department of Occupational Therapy, Soonchunhyang University, Asan-si 31538, Republic of Korea; wjdgkssk122@sch.ac.kr; 2 Department of ICT Convergence, Soonchunhyang University, Asan-si 31538, Republic of Korea; choidabin08@sch.ac.kr; 3 Sensory Integration toward Social and Occupational Being, Seoul 04061, Republic of Korea; meesun89@gmail.com (M.K.); jiseokyeon@gmail.com (S.J.); 4 Department of Psychiatry, Samsung Medical Center, Sungkyunkwan University School of Medicine, Seoul 06351, Republic of Korea

**Keywords:** BOT-2, short form, Korean children, school-age children, psychometric study, validity

## Abstract

The Bruininks–Oseretsky Test of Motor Proficiency Second Edition (BOT-2) is the most common motor assessment in Korea. The BOT-2–Short Form (SF) is preferred over the complete form (CF) in settings with limited time. The present study aimed to assess the validity of the BOT-2 SF in Korean school-age children. First, we verified that the BOT-2 SF reflects developmental changes in motor skills. Second, we compared the BOT-2 SF scores to those of the BOT-2 CF. A total of 283 Korean school-age children performed the BOT-2. The differences in the BOT-2 SF point according to age group (7 years, 8–9 years, and 10–12 years) were analyzed. A correlation analysis of the standard scores between the BOT-2 SF and CF was conducted. The sensitivity and specificity of the BOT-2 SF were calculated in reference to its CF. Overall, the BOT-2 SF point scores increased with age. The correlation between the total scores of the BOT-2 SF and CF was strong. The BOT-2 SF had a sensitivity of 83% and specificity of 92%. This study has demonstrated the validity of the BOT-2 SF in Korean school-age children. The BOT2 SF can be useful in screening Korean school-age children with motor skills problems.

## 1. Introduction

Children gradually acquire the ability to move their bodies precisely and appropriately through psychomotor development. Motor proficiency is closely related to perceptual, cognitive, and socioemotional functions [1,2,3,4]. Motor proficiency in childhood is linked to competence in other developmental tasks, including academic achievement [5,6] and peer relationships [7]. Children with good motor abilities are more likely to be successful in daily activities, whereas those with poor motor skills are less likely to meet task demands.

Psychomotor problems have been frequently observed in children with neurodevelopmental disorders, such as autism spectrum disorder [8], attention-deficit/hyperactivity disorder [9], and learning disorders [10]. Particularly, developmental coordination disorder (DCD) is characterized by core deficits in motor coordination [11]. These deficits are associated not only with physical difficulties, but also with psychological and social disturbances [12,13]. Therefore, assessing motor proficiency during childhood is important to identify motor difficulties and provide appropriate interventions.

The Bruininks–Oseretsky Test of Motor Proficiency Second Edition (BOT-2) [14] is the most common motor assessment tool used by occupational therapy practitioners for Korean children [15]. The BOT-2 is a standardized test for assessing motor skills in children and youth aged 4–21 years. There are two forms of the BOT-2: the complete form (CF) and the short form (SF). The CF provides comprehensive information on motor function, and is recognized as the gold standard for evaluating motor performance at developmental stages [16,17]. Of the 53 items comprising the CF, 14 items were selected to constitute the SF. The SF can be applied to screen for probable motor deficits to determine further evaluation. Although the SF has been frequently used by Korean occupational therapy practitioners because of its short assessment time [18,19,20], its validity in Korean children has not been investigated.

In the BOT-2 development study, the SF showed adequate reliability and validity comparable to that of the CF [14]. The SF showed internal consistency of 0.71 to 0.91, test–retest reliability of 0.84 to 0.91, and inter-rater reliability of 0.97 to 0.98. Children with DCD received lower SF scores than typically developing children. The validity of the SF has been investigated since publication. Previous studies have shown that the SF point scores increase with age [21,22], indicating that the SF can assess developmental changes. The relationship between the SF and other motor assessments such as the Körperkoordinationstest für Kinder [21,23] and the McCarron Neuromuscular Development Assessment [24,25] demonstrated convergent validity.

A recent study investigated the compatibility between the BOT-2 SF and its CF in German school-age children [26]. This previous study reported that the total standard scores of the SF were strongly correlated with those of the CF. In the same study, analyses of the SF screening accuracy based on the CF revealed that the SF had high sensitivity but low specificity (84% and 42.9%, respectively). The poor specificity of the SF for German children could be attributed to the participating children who scored lower than the United States normative sample.

Motor performance can be influenced by culture [27,28,29]. For example, East Asian American children perform better in terms of fine motor skills than European American children [30]. Chinese children have higher levels of fundamental movement skills than England’s children, which could be attributed to different engagement opportunities in movement-related activities [31]. These findings suggest that the validity of motor assessment is culture-specific; findings from one culture do not necessarily apply to other cultures.

In the present study, we examined the validity of the BOT-2 SF in Korean school-age children. First, we investigated whether Korean children’s motor skills rated on the SF increase with age. If the BOT-2 SF includes items that measure developmental changes in motor skills, older children would score higher than younger children. Second, we examined the correlation between the SF and the CF scores in Korean children. If the SF is comparable to that of the CF, a strong correlation should exist between the two forms. Third, to determine the usefulness of the SF as a screening tool in Korean children, we calculated the sensitivity and specificity of the SF in reference to the CF. We hypothesized that the SF would reflect the motor development of Korean children, and show a strong correlation with the CF scores, which would support the use of the SF to identify Korean children with probable DCD.

## 2. Materials and Methods

### 2.1. Participants

This study included 283 school-age Korean children, 146 boys and 137 girls (M age = 9.1 years, SD = 1.66). Children were recruited from communities in Seoul (*n* = 146), Gyeonggi-do (*n* = 124), Incheon (*n* = 7), and Daejeon (*n* = 6), South Korea. The participants were part of a general population of these communities, which also included children with attention-deficit/hyperactivity disorder (*n* = 5), language disorder (*n* = 3), and developmental delay (*n* = 1). We excluded children who did not complete all test items or had physical difficulties (e.g., cerebral palsy). Data were collected individually (*n* = 38) or in elementary schools (*n* = 245). Fourteen classes were recruited as homeroom teachers. Each class had about 18 students, most of whom were tested by about 8 occupational therapists. Participants were divided into three age groups (7 years, 8 to 9 years, and 10 to 12 years of age) based on the Developmental Coordination Disorder Questionnaire 2007 [32], which divides the scoring system into 7 years and younger, 8 to 9 years, and 10 years and older. The G*Power presented the total sample size of 84, assuming an effect size of 0.25, an alpha level of 0.05, and a power of 0.95 for the MANOVA. The number of participants in this study was greater than the suggested sample size. The sample size of this study was similar to those of previous studies, which had between 70 and 106 participants in each age group [33,34].

This preliminary cross-sectional study was part of the Korean BOT-2 standardization project. The study was approved by the Institutional Review Board of the Soonchunhyang University (protocol code 1040875-201805-SB-016 and 07/23/2018), and all participants provided informed consent. Data were collected between November 2018 and June 2022. The BOT-2 was administered using a procedure similar to that described by Yoon et al. [17].

### 2.2. Measures

#### Bruininks–Oseretsky Test of Motor Proficiency Second Edition (BOT-2)

The BOT-2 is a standardized assessment tool of motor skills in individuals aged 4–21 years [14]. The BOT-2 has two forms, the CF, composed of 53 items, and the SF, composed of 14 out of the 53 items. For each item, a raw score corresponds to a point score. In the SF, the point scores are directly summed into a total point score and then converted to a standard score. In the CF, point scores are sequentially organized and transformed into eight scale scores of fine motor precision (FMP), fine motor integration (FMI), manual dexterity (MD), bilateral coordination (BC), balance (B), running speed and agility (RSA), upper-limb coordination (UC), and strength (S), which consist of four motor area composites of fine manual control with FMP and FMI, manual coordination with MD and UC, body coordination with BC and B, and strength and agility with RSA and S, as well as the total motor composite. The standard scores for the total of the SF, and the motor area and total motor composites of the CF, have a mean of 50 and an SD of 10. Standard scores can be categorized as 70 or greater (well above average), 60–69 (above average), 41–59 (average), 31–40 (below average), and 30 or less (well below average). Internal consistency reliability for the BOT-2 development study was high for the CF (0.93 to 0.97) and respectable to very good for the SF with knee push-ups (0.75 to 0.91). According to our study’s data, internal consistency reliability was very good for the CF (0.90) and minimally acceptable for the SF with knee push-ups (0.69) [35,36].

### 2.3. Data Analysis

The participants were divided into three age groups: 7 years, 8–9 years, and 10–12 years [17,21,32]. The SF data were derived from the CF data. For the SF, descriptive statistics for each item and total point scores in each age group were calculated. To identify the change in motor performance according to age, the SF point scores were analyzed using a multivariate analysis of variance (MANOVA) with age group as a between-participants factor, followed by the post-hoc Bonferroni test. We used IBM SPSS Statistics, version 22.0 (IBM Corp., Armonk, NY, USA) for statistical analyses.

To examine the association between the SF and the CF, we performed a Pearson correlation analysis on the standard scores between the SF totals and the CF motor area and total motor composites. Correlation coefficients were calculated for each age group and for all groups. We interpreted a correlation coefficient of 0.7 and above as strong, 0.3 to 0.7 as moderate, and below 0.3 as weak [37].

To examine the usefulness of the SF as a screening tool, the sensitivity and specificity of the SF using the CF as a reference were calculated. Children in below average or lower categories of the CF total motor composite were assumed to have probable DCD [17,38]. Sensitivity was considered as the proportion of children in the below average or lower categories of the SF among children who were also in the below average or lower categories of the CF. Specificity was considered as the proportion of children in the average or higher categories of the SF among children who were also in the average or higher categories of the CF. For a screening test, a sensitivity ≥ 80% and a specificity ≥ 90% are preferable [39,40].

## 3. Results

A total of 283 children were divided into three age groups: 7 years (M age = 7.62 years, SD = 0.25; 55 boys and 47 girls), 8–9 years (M age = 8.57 years, SD = 0.57; 47 boys and 44 girls) and 10–12 years (M age = 11.29 years, SD = 0.80; 44 boys and 46 girls). Table 1 shows the mean and standard deviation of the SF point scores for each age group.

The MANOVA results show that age had a significant effect on the SF total point scores (Table 2). The SF total point score increased significantly with age. The 10–12 age group had a higher total mean than the 7- and 8–9 age groups. The 8–9 age group also had a higher total mean than the 7 age group. The point scores of 10 out of the 14 items of the SF significantly increased with age. However, there was no age difference in the point scores for the following four items: “FMI2. copying a square”, “BC6. tapping feet and fingers—same sides synchronized”, “B2. walking forward on a line”, and “B7. standing on one leg on a balance beam—eyes open”.

The descriptive statistics for the standard scores of the BOT-2 SF and CF are presented in Table 3. The means of the BOT-2 SF and CF total standard scores for all groups are 51.11 and 52.42, respectively.

**Figure 1:**
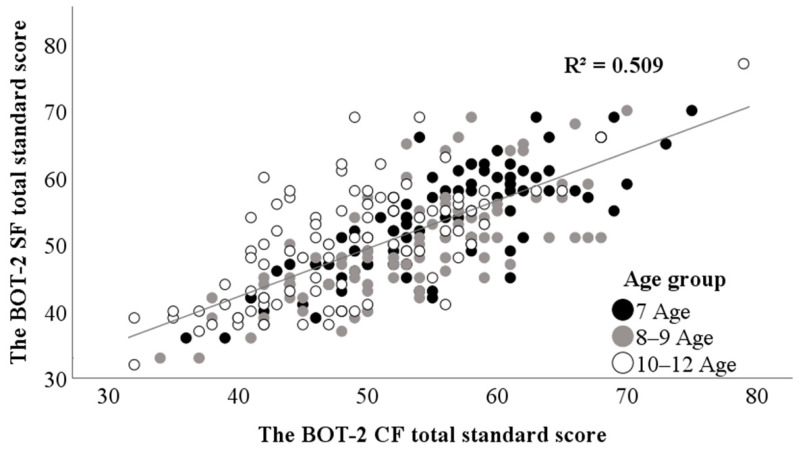
The scatter plot and a trend line between the BOT-2 SF total standard scores and the CF total standard scores. BOT-2: Bruininks–Oseretsky Test of Motor Proficiency Second Edition. SF: Short form. CF: Complete form.

Table 4 presents correlation results of standard scores between the SF total and the CF motor area and total motor composites. For all participating children, the correlation between the SF total and the CF total was strong (r = 0.71). The relationship was the strongest in the 7 age group, and the strength of correlation decreased with age (Figure 1). Regarding the relationship between the SF total and the CF motor areas for all participating children, the SF total was strongly correlated with “strength and agility” area scores, moderately correlated with “manual coordination” area scores, and weakly correlated with “fine manual control” and “body coordination” area scores. The correlations between the SF score and the CF scores of “fine manual control” and “body coordination” decreased with age; these were weak to moderate in the 7 age group, weak in the 8–9 age group, and not statistically significant in the 10–12 age group.

We calculated the sensitivity and specificity of the SF for identifying children with DCD in reference to the CF. The SF correctly classified 15 of the 18 children with probable DCD, and 245 of the 265 children without probable DCD. The BOT-2 SF had a sensitivity of 83% and specificity of 92%, with an overall accuracy of 91.9%.

## 4. Discussion

The present study investigated the validity of the BOT-2 SF for Korean school-age children. The SF total point scores increased with age, indicating that the SF can measure the development of motor skills. The SF total standard score was strongly correlated with the CF total standard score, demonstrating that the SF was comparable to the CF. Furthermore, the SF total standard score successfully identified children with or without probable DCD, suggesting that the SF can be applied as a useful screening tool for DCD in Korean school-age children. This study demonstrates additional psychometric evidence of the cross-cultural use of the SF [26,41].

For the BOT2-2 SF total point score, older children scored higher than younger children. At the individual item level, point scores for each item increased differently with age. Among the 14 items, 10 item scores were affected by age, whereas 4 items did not show any significant change. These four items were “FMI2. copying a square”, “BC6. tapping feet and fingers—same sides synchronized”, “B2. walking forward on a line”, and “B7. standing on one leg on a balance beam—eyes open”. These items might not differentiate motor skills in Korean children aged 7–12 years [33,34]. Previous studies of the BOT-2 SF have reported no significant correlation of FMI2, BC6, B2, and B7 items with the category scores in which the items are included [42,43,44], suggesting that these items may not be valid for assessing the motor domain. Most children of this study scored the maximum on the following items: “FMI2. copying a square”, “BC6. tapping feet and fingers—same sides synchronized”, and “B2. walking forward on a line”. The motor skills involved in these items appear to be acquired before the age of 7. For example, most Korean 5-year-old children can copy a square [45]. For “B7. standing on one leg on a balance beam—eyes open”, children did not achieve the maximum point, yet there was no significant change in the point score with age. Balance ability increased sharply at the preschool age, reaching a saturation point at age 7 in the BOT-2 development study [14]. Although some items did not show significant differences by age, the majority of items and the total point score on the SF reflected the development of motor skills in Korean school-age children.

The BOT-2 SF total standard scores had a strong correlation with its CF total standard scores (r = 0.71), which is consistent with a previous finding in German children (r = 0.76) [26]. The SF score was also correlated with the motor area score of the CF. The correlation between the SF and the CF four motor areas was high in the order of “strength and agility” (r = 0.79), “manual coordination” (r = 0.69), “fine manual control” (r = 0.26), and “body coordination” (r = 0.24). The correlations of the SF with “fine manual control” and “body coordination” areas were weaker than those with “strength and agility” and “manual coordination”, which is aligned with the German study [26]. These weak correlations may be because undiscriminating items (FMI2, BC6, B2, and B7) of the SF are included in the “fine manual control” and “body coordination” areas. The correlation between the SF and these two motor areas was weak to moderate in the 7 age group, weak in the 8–9 age group, and insignificant in the 10–12 age group. These results imply that the BOT-2 SF has limitations in representing “fine manual control” and “body coordination” in older school-age children. Meanwhile, the correlation of the SF with “manual coordination” and “strength and agility” was high in the 10–12 age group, suggesting that the BOT-2 SF items may reflect these motor areas better in older children. These results suggest that the items comprising the BOT-2 SF would have differential discriminatory power depending on age.

This study provides clinical implications related to the application of the BOT-2 SF. When using the SF in practice, it is important to be aware that the SF can only provide general information about motor skills, and has weak correlations with “fine manual control” and “body coordination”. To measure various aspects of motor proficiency, more comprehensive assessments such as the CF need to be applied.

The use of the BOT-2 SF has been suggested as a screening tool to identify children with motor impairments [16,46]. To examine the diagnostic accuracy of a screening tool, it is recommended to compare it to a comprehensive reference standard [47], such as the Diagnostic and Statistical Manual of Mental Disorders [11]. Further studies could investigate the predictive validity of the SF with a clinical diagnosis of DCD.

The BOT-2 SF showed an adequate sensitivity of 83%, indicating that it could be a useful tool for screening children with probable DCD. In addition, the assessment showed a preferable specificity of 92%, suggesting that the BOT-2 SF could accurately classify children without probable DCD. This specificity for Korean school-age children is inconsistent with the low specificity for German children (42.9%) [26]. This discrepancy between the two studies can be attributed to differences in the mean total standard scores. The low specificity of the German study indicates that many children without DCD in the CF were classified as having DCD in the SF. The mean for the standard score in the BOT-2 development study was 50, whereas the mean scores for the SF and the CF in the German study were 45.87 and 47.57, respectively. Furthermore, the mean score of the SF was lower than that of the CF. Because of these statistical characteristics of lower scores, many German children without motor problems based on the CF might be classified as having probable DCD based on the SF. In contrast, the means in Korean school-age children were 51.11 for the SF and 52.42 for the CF, closer to 50, allowing for better specificity in the present study. In summary, the BOT-2 SF showed good specificity and sensitivity for screening possible motor problems in Korean school-age children.

The present study has some limitations that require further research. First, the BOT-2 can be used for assessment in individuals aged 4–21 years. However, the findings of this study were obtained from Korean children aged 7–12 years. These results should be interpreted with caution in the context of age and culture. Future studies are needed to investigate the validity of the SF in preschoolers and youth. Second, this study was based on a community sample. To evaluate the clinical utility of the SF, children diagnosed with DCD should be included in future studies. Third, most data were collected in Seoul and Gyeonggi-do, which account for approximately 45 percent of the Korean child population. Only a small number of children were recruited from Incheon and Daejeon. Future studies need to collect data from across the country so that the data can be more representative. Fourth, participants were divided into three groups consisting of one or more ages, with 90–102 children in each group. Meanwhile, dividing children by each age helps to examine detailed developmental changes in motor skills. Future studies need to have sufficient samples of children across different age groups. Fifth, confounding variables such as socioeconomic status and body mass index were not obtained and controlled in the analysis. However, because most of the data in this study were collected from classrooms where students were randomly and equally assigned, the risk of bias due to confounding variables was low. Future studies need to consider confounding variables to investigate motor functions in Korean children.

## 5. Conclusions

The present study demonstrated the validity of the BOT-2 SF in assessing the overall motor proficiency of Korean school-age children. The SF can measure developmental changes in motor skills and provide results comparable to the CF. The SF can successfully distinguish children with and without probable DCD. In conclusion, the BOT-2 SF can be used as a screening tool for motor problems in Korean school-age children to determine the need for further evaluation.

## Figures and Tables

**Table 1:** Mean and standard deviation of the BOT-2 point scores.

	7 Age Group	8–9 Age Group	10–12 Age Group
	(*n* = 102)	(*n* = 91)	(*n* = 90)
SF total point score	63.99 (6.11)	66.64 (6.15)	72.67 (6.11)
CF total point score	227.75 (19.86)	239.42 (18.55)	259.88 (19.97)
FMP3. Drawing lines through paths—crooked	6.71 (0.68)	6.82 (0.46)	6.93 (0.29)
FMP6. Folding paper	6.69 (0.84)	6.75 (0.59)	6.96 (0.26)
FMI2. Copying a square	4.88 (0.32)	4.90 (0.30)	4.87 (0.34)
FMI7. Copying a star	3.48 (1.03)	3.69 (0.88)	4.13 (0.78)
MD2. Transferring pennies	6.49 (1.11)	6.85 (1.11)	7.50 (1.08)
BC3. Jumping in place—same sides synchronized	2.90 (0.41)	2.98 (0.15)	3.00 (0.00)
BC6. Tapping feet and fingers—same sides synchronized	3.99 (0.10)	3.95 (0.43)	3.99 (0.11)
B2. Walking forward on a line	4.00 (0.00)	4.00 (0.00)	3.98 (0.21)
B7. Standing on one leg on a balance beam—eyes open	3.45 (0.86)	3.52 (0.92)	3.63 (0.69)
RSA3. One-legged stationary hop	8.30 (0.88)	8.34 (0.82)	9.03 (1.17)
UC1. Dropping and catching a ball—both hands	3.89 (1.28)	4.43 (0.87)	4.69 (0.91)
UC6. Dribbling a ball—alternating hands	3.54 (1.82)	4.31 (1.87)	5.47 (1.78)
S2. Knee push-ups	2.34 (1.81)	2.42 (1.94)	3.58 (2.03)
S3. Sit-ups	3.32 (1.64)	3.69 (1.81)	4.91 (2.00)

BOT-2: Bruininks–Oseretsky Test of Motor Proficiency Second Edition. SF: Short form. CF: Complete form. FMP: Fine motor precision. FMI: Fine motor integration. MD: Manual dexterity. BC: Bilateral coordination. B: Balance. RSA: Running speed and agility. UC: Upper-limb coordination. S: Strength.

**Table 2:** Comparison of the BOT-2 SF point scores by age group.

	F	Mean Difference between Age Groups		
		7 vs. 10–12	7 vs. 8–9	8–9 vs. 10–12
Total point score	49.67 **	−8.68 **	−2.65 **	−6.03 **
FMP3. Drawing lines through paths—crooked	4.70 **	−0.23 **	−0.12	−0.11
FMP6. Folding paper	4.79 **	−0.27 **	−0.06	−0.21
FMI2. Copying a square	0.26	0.02	−0.02	0.03
FMI7. Copying a star	12.67 **	−0.65 **	−0.21	−0.44 **
MD2. Transferring pennies	20.56 **	−1.01 **	−0.36	−0.65 **
BC3. Jumping in place—same sides synchronized	3.80 *	−0.10 *	−0.08	−0.02
BC6. Tapping feet and fingers—same sides synchronized	0.93	0.00	0.05	−0.04
B2. Walking forward on a line	1.07	0.02	0.00	0.02
B7. Standing on one leg on a balance beam—eyes open	1.16	−0.18	−0.07	−0.12
RSA3. One-legged stationary hop	16.86 **	−0.73 **	−0.04	−0.69 **
UC1. Dropping and catching a ball—both hands	14.57 **	−0.80 **	−0.54 **	−0.26
UC6. Dribbling a ball—alternating hands	26.88 **	−1.93 **	−0.77 *	−1.16 **
S2. Knee push-ups	11.96 **	−1.23 **	−0.07	−1.16 **
S3. Sit-ups	19.64 **	−1.59 **	−0.37	−1.22 **

* *p* < 0.05; ** *p* < 0.01, BOT-2 SF: Bruininks–Oseretsky Test of Motor Proficiency Second Edition Short Form. FMP: Fine motor precision. FMI: Fine motor integration. MD: Manual dexterity. BC: Bilateral coordination. B: Balance. RSA: Running speed and agility. UC: Upper-limb coordination. S: Strength.

**Table 3:** Mean and standard deviation of standard scores on the BOT-2 SF and CF.

	7 Age Group	8–9 Age Group	10–12 Age Group	All Groups
SF total	53.18 (7.79)	50.04 (7.97)	49.83 (8.79)	51.11 (8.30)
CF total	55.24 (7.58)	53.31 (7.54)	48.33 (8.04)	52.42 (8.22)
Fine manual control	58.02 (6.68)	54.53 (6.22)	49.84 (7.48)	54.30 (7.57)
Manual coordination	51.12 (8.47)	50.51 (8.42)	46.93 (8.31)	49.59 (8.57)
Body coordination	49.04 (7.10)	49.53 (8.61)	45.79 (8.34)	48.16 (8.14)
Strength and agility	57.82 (8.98)	55.97 (8.55)	53.80 (8.14)	55.95 (8.71)

BOT-2: Bruininks–Oseretsky Test of Motor Proficiency Second Edition. SF: Short form. CF: Complete form.

**Table 4:** Correlation coefficients between the BOT-2 SF and CF.

	7 Age Group	8–9 Age Group	10–12 Age Group	All Groups
CF total	0.80 **	0.70 **	0.67 **	0.71 **
Fine manual control	0.25 *	0.23 *	0.17	0.26 **
Manual coordination	0.69 **	0.68 **	0.70 **	0.69 **
Body coordination	0.35 **	0.25 *	0.12	0.24 **
Strength and agility	0.80 **	0.71 **	0.84 **	0.79 **

* *p* < 0.05; ** *p* < 0.01. BOT-2: Bruininks–Oseretsky Test of Motor Proficiency Second Edition. SF: Short form. CF: Complete form.

## Data Availability

The data presented in this study are available on request from the corresponding author due to the privacy of children.
